# Flowering phenology in a *Eucalyptus loxophleba* seed orchard, heritability and genetic correlation with biomass production and cineole: breeding strategy implications

**DOI:** 10.1038/s41598-020-72346-3

**Published:** 2020-09-17

**Authors:** Beren Spencer, Richard Mazanec, Amir Abadi, Mark Gibberd, Ayalsew Zerihun

**Affiliations:** 1 School of Molecular and Life Science, Curtin University, GPO Box U1987, Perth, WA 6845 Australia; 2 Department of Biodiversity, Conservation and Attractions, Kensington, WA 6151 Australia

**Keywords:** Plant breeding, Plant domestication, Plant reproduction, Heritable quantitative trait

## Abstract

Reproductive synchronicity within a seed orchard facilitates gene exchange and reduces self-fertilisation. Here we assessed key flowering traits, biomass and foliar 1,8-cineole concentrations of *Eucalyptus loxophleba* (subsp. *lissophloia* and *gratiae*) in an open-pollinated seed orchard. Monthly flowering observations were made on 1142 trees from 60 families and nine provenances across 2 years. The percentage of trees flowering in both years was similar at 87%. There were differences between provenances and families within provenances for flowering traits, biomass and 1,8-cineole and interactions between provenances and year for flowering traits. Heritability of start and end flowering, and 1,8-cineole were high to moderate ($\hat{h}^{2}$ = 0.75–0.45) and duration of flowering, propensity to flower and biomass estimates were moderate to low ($\hat{h}^{2}$ = 0.31–0.10). Genetic and phenotypic correlations between flowering traits were high (*r*_*g*_ = 0.96–0.63 and *r*_*p*_ = 0.93–0.34) except between duration and end of flowering. The correlations were weaker between flowering traits and biomass or 1,8-cineole. ‘Dual flowering’, when trees underwent two reproductive cycles in a year, was responsible for out-of-phase flowering and those with low biomass and 1,8-cineole concentration should be removed from the breeding programme to hasten selection for desirable traits.

## Introduction

In southwest Western Australia, as elsewhere in Australia, the large scale conversion of native vegetation to agricultural land was followed by extensive salinity problems^1,2^. In the early 1990s, a research program was initiated to identify multi-purpose perennial crops that could mitigate the salinity problems while providing additional economic benefit when integrated with the annual crop based farming systems of the region^3^. The research identified mallee eucalypts as the preferred candidates due to their capacity to coppice after short-cycle harvest^4–7^. While the initial selection of mallee species focussed on foliar 1,8-cineole concentration (hereafter referred to as cineole), for large-scale bio-renewable feedstock^8–11^, subsequently, biomass yield emerged as an additional selection criterion as the opportunity for carbon sequestration and bioenergy became prospective during the late 1990s^12–14^.

Two species, *Eucalyptus loxophleba* subsp. *Lissophloia* L.A.S. Johnson & K.D. Hill, hereafter referred to as E_liss_, and *E. loxophleba* subsp. *gratiae* Brooker, hereafter referred to as E_grat_ were selected for development given their high concentrations of foliar cineole, fast growth rates and prominence in native woodlands of the southwest of Western Australia^15,16^. Successful development of improved tree crops, with multiple desirable traits, presents a challenge with limited understanding of key genetic parameters, such as heritability, genetic and phenotypic correlations. Thus, in 1993 a breeding programme was initiated; and by 2002, 11 *E. loxophleba* trials had been established using progeny from a total of 78 parents selected in the wild for high cineole content^17,18^. The trials were assessed for foliar cineole concentration and biomass then thinned to produce genetically improved seed for agroforestry plantings. This study assessed one of these open-pollinated seed orchards for key flowering traits, cineole and biomass yield with the aim of facilitating production of improved seed to enhance the potential commercial viability of these species as tree crops.

The timing of key reproductive events is crucial for outcrossing, and in eucalypts barriers that restrict pollen dispersal can result in self-pollination and reduced fitness^19–22^. Open-pollinated seed orchards, without any reproductive isolation could facilitate panmixia resulting in panmictic equilibrium. A shift from panmictic equilibrium (e.g. due to asynchrony in flowering time) may lead to unbalanced contributions of genetic material from individual parents to the next generation and reproductive isolation^23^. Several studies have shown that time of anthesis in eucalyptus is under genetic control, resulting in different flowering times between provenances and families^24–27^. Therefore, understanding the reproductive phenology of a seed orchard can enable culling reproductively isolated trees, families or provenances thereby increasing the overall level of outcrossing in the orchard.

Synchronicity of flowering within a seed orchard also enables transmission of favourable genes or traits in a breeding programme. For instance, foliar cineole concentration for E_liss_ has a high narrow sense heritability of 0.53 ± 0.07^18^. Fully exploiting traits with high heritability relies on outcrossing (synchronised flowering) within a seed orchard. Padovan et al*.*^28^ identified 12 single nucleotide polymorphisms for *E. loxophleba* that are associated with terpene production including two for cineole. However, when selecting for a breeding trait, unfavourable genetic correlation between traits could compromise gains for the second trait. For instance, with *E. camaldulensis*, there is a moderate negative correlation between total monoterpenes (including cineole) and biomass^29^. Understanding the correlations between key flowering traits, biomass and foliar cineole concentration will reduce unfavourable bias from breeding selections.

The flowering phenology of several eucalypt species are documented, for example *E. regnans*^30^ and *E. globulus*^24^, but only general observations of flowering periods in natural populations have been made for E_liss_ and E_grat_. Records of the timing of flowering activity for E_liss_ indicate some variation including September to February^31^ and August to October^32^. Similarly, E_grat_ has been documented to flower between September and December^31^ and from October to November^32^. Bell^33^ monitored flowering in three E_liss_ families in a seed orchard near Collie, Western Australia, where peak flowering occurred from November to December, however some individuals flowered as early as June/July. Observations in E_liss_ and E_grat_ seed orchards and in wild populations indicated that flowering can be observed at almost any time of the year (B. Spencer and W. O’Sullivan personal observations).

The timing and duration of anthesis for a species determines its capacity to breed within a plantation but also with neighbouring populations of closely related species. Genetic pollution by pollen dispersal has been identified as an important conservation threat that can lead to extinction^34^. This is particularly so in the *Eucalyptus* genus due to highly mobile pollen and weak reproductive barriers^35^. Further, the considerable interest in tree planting on farms has facilitated pollen flow between formerly isolated species resulting in exotic eucalypt hybrids^36,37^ and the *E. loxophleba* group is known to be a prolific pollinator. For example, an exotic E_liss_ planting has pollinated over half of a remnant *E. loxophleba* ssp. *supralaevis* population, with the exotic pollen travelling up to 1940 m from its source^38^. *Eucalyptus loxophleba* has been recorded as hybridising with *E. kruseana*^39^, *E. wandoo*^40^ and *E. absita*^41^ where the species naturally co-exist. Additionally, the Western Australian Herbarium database lists hybrids of the various subspecies of *E. loxophleba* with *E. accedens, E. astringens, E. blaxellii, E. erythronema, E. occidentalis, E. orthostemon, E. rudis, E. spathulata* and *E. victrix*^42^.

This work examines the flowering phenology of E_liss_ and E_grat_ in a single orchard and determines:

- the variation between and within subspecies, provenances and families to identify out of phase provenances and families under a common environment setting; and
- the heritability and genetic parameters of key flowering traits, biomass and foliar cineole.

It is expected that knowledge of genetic control and heritability of the various traits considered here, and culling of those which flower outside of the peak flowering period from the seed orchard will speed up production of seeds with desired traits and support commercial viability of mallee as bioenergy tree corps.

## Methods

### Study site

The study site was located approximately 200 km south east of Perth near Toolibin Lake (32.88°S, 117.62°E). The average rainfall is approximately 400 mm and falls mainly between May and September.

**Figure 1:**
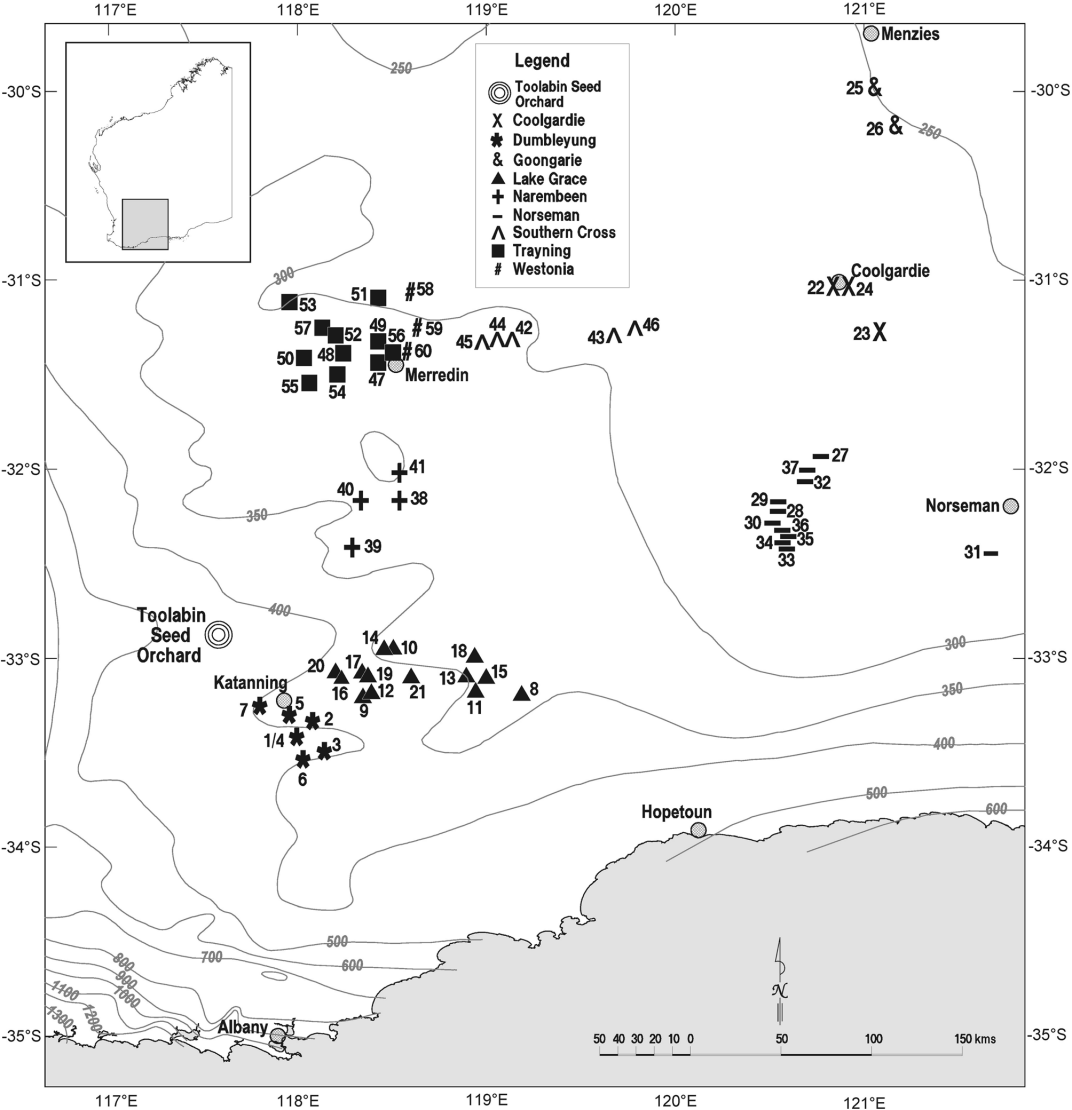
The location of the 60 parent trees from the *Eucalyptus loxophleba* seed orchard and their provenances. Circles represent key population centres and large triple circle is the location of the seed orchard. The eastern E_liss_ region comprises Goongarrie, Coolgardie and Norseman. The western E_liss_ region comprises Westonia, Trayning, Southern Cross and Narembeen. The Egrat subsp. has two provenances, Lake Grace and Dumbleyung. This map was generated using MicroStation Version 8i. Bentley Systems Incorporated, 2006 https://www.bentley.com/en/products/brands/microstation.

The seed orchard, containing both E_liss_ and E_grat_ was planted in 1999 and the population contains 60 families from nine provenances and three broad regions encompassing the full natural distribution of these taxa (Table 1, Fig. 1). Here a family is defined as the sexually produced open-pollinated progeny from a wild parent tree and a provenance refers to the progeny of wild trees from a known geographic range. The distribution of E_liss_ is much wider than E_grat_ and to reflect this, seven of the nine provenances were E_liss_ (Fig. 1). The 60 families were planted in six-tree row plots in a randomised row-column design with 20 replicates. The orchard was thinned in 2005 from 7200 trees to 1142 based on breeding values for foliar cineole concentration and above ground biomass. After thinning, all 60 families and nine provenances were still represented in the seed orchard with the number of trees retained in each family ranging from 14 to 22.

**Table 1:** Descriptions of regions, provenances, and climate of locations from where the two subspecies of *Eucalyptus loxophleba* for the seed orchard planting were sourced. The number of families in each provenance and individual trees assessed for flowering traits are also described. Climatic data and elevation which were obtained from SILO dataset^43^ from 1985 to 2015.

Region	Provenance	No families	No trees assessed	Mean maximum temperature (°C)	Mean minimum temperature (°C)	Mean annual rainfall (mm)	Mean annual pan evaporation (mm)	Elevation (m)
E_grat_ South	Dumbleyung	7	133	22.9	9.9	340.5	1674.1	341
E_grat_ South	Lake Grace	14	274	23.5	10.0	330.3	1798.4	329
E_liss_ East	Coolgardie	3	53	25.1	11.2	306.4	2412.7	469
E_liss_ East	Goongarrie	2	39	26.5	12.7	275.1	2698.4	415
E_liss_ East	Norseman	11	217	24.6	10.4	302.4	2114.2	375
E_liss_ West	Narembeen	4	78	24.2	10.0	370.1	1931.5	457
E_liss_ West	SouthernCrs	5	90	25.4	11.4	317.6	2167.7	400
E_liss_ West	Trayning	11	208	25.7	11.6	321.8	2235.2	378
E_liss_ West	Westonia	3	50	25.5	11.0	335.3	2264.2	354

### Floral assessments

Floral assessments were completed approximately every 4 weeks from May 2012 to January 2013 and from February 2014 to January 2015. To carry out these assessments the canopy of each tree was scanned for presence of reproductive activity from the ground using 8 × 40 binoculars in teams of two to reduce assessor bias. In addition, several times a day, observer teams were calibrated with each other to further reduce bias.

Each tree in the seed orchard was assessed for the three stages of reproduction: immature buds; mature buds; and flowering. Firstly, the immature buds were assessed following the shedding of the involucral bracts. This was separated into three phases: a score of one was given when the involucral bracts had just shed but the buds were still clustered together; a score of two indicated clear separation between the buds; a score of three was given when the operculum was visible and buds were approaching mature size. If a tree had more than one of these phases present during any one assessment, the different phases were recorded. In the 2012 assessment, it was noted that many individual trees had two reproductive events within the year. In the 2014 assessment, where these separate events were observed within a tree, they were recorded separately. These are referred to as reproductive flushes.

After the buds had matured, the bud density was assessed on a scale of one to four. A rank of four was given to trees with a dense crop (mature buds, flowers and capsules post-anthesis) distributed evenly throughout the tree. A score of three was given to trees with an average crop density, or a dense crop unevenly distributed throughout the canopy. A score of two was given to trees with a sparse crop and a score of one to trees with a crop too sparse to accurately assess.

Finally, as flowering matured, the percentage of buds, active flowers and spent flowers were estimated from the full canopy of each tree. In the 2014 assessment, each reproductive flush was recorded separately. Different flushes were discernible because there was a physical gap between the reproductive flushes which were usually separated by vegetative growth and the newer buds were smaller and brighter green. Flowers were determined as active when the operculum had been shed and white or cream anthers were evident. Flowers were classified as spent when the anthers were desiccated, brown or absent.

### Flowering criteria

Where a bud density score of one was observed, that reproductive flush was eliminated from the analysis. These flushes were sparse and scores fluctuated widely between assessments. This appeared to be due to loss of reproductive structures through natural abortion or predation processes. These flushes also tended to receive inflated flowering scores because flowers are more prominent than buds on a sparse crop. We defined flowering as a phase when at least two percent of the buds in a reproductive phase were in flower with fresh anthers present. A score of 1% was used to represent few extant flowers to assist the team in the following assessment to indicate that the tree had commenced flowering. Additionally, a tree was defined as flowering when more than 5% of its pre-flowering buds had progressed past anthesis between assessments. This was rarely seen during autumn and winter assessments, but was on occasions in spring and early summer, when flowering proceeded more rapidly.

### Biomass assessment

Two biomass assessments were taken for individuals within this seed orchard. The first assessment was taken pre-thinning in autumn 2004 by measuring the Crown Volume Index (CVI), as described in Spencer et al*.*^5^, of each of the 5372 healthy trees in the orchard. Briefly, CVI is the measurement of the height and two perpendicular crown widths expressed as m^3^.

The stem basal area of the remaining trees post-thinning (1163 trees) was assessed in February 2014. The stems of each tree were measured with a diameter tape approximately 10 cm above the ground. Loose and fibrous bark was removed and burls and buttressing associated with the lignotubers were avoided. A single diameter estimate was obtained by calculating the Equivalent Diameter near Root Collar (EDRC) as specified in Chojnacky and Milton^44^ using the formula:

1 $$ EDRC = \sqrt {\mathop \sum \limits_{i = 1}^{{\text{n}}} {\text{drc}}_{{\text{i}}}^{2} } $$

where *n* is number the number of stems *drc* is individual diameter of each stem. General E_liss_ species allometric equations were applied to the natural log of CVI and stem diameter to convert to dry biomass as specified in Spencer et al*.*^5^ using the back-transformation correction methodology as outlined by Baskerville^45^.

### Cineole assessment

Leaf samples were collected in autumn 2004 from 5223 healthy trees in the seed orchard. For each tree, cineole was extracted from 3 g leaf samples in 50 mL ethanol for ≥ 4 weeks and analysed using HP5890A gas chromatograph as detailed in Mazanec et al*.*^18^.

### Heat sum

Heat sum was calculated by averaging the maximum and minimum daily temperatures above a base temperature below which an organism will not develop^46^. Climatic data was obtained through SILO from interpolated dataset^43^. We used a base temperature of 5 °C which was determined for *E. globulus*^47^ and used in eucalypt flowering studies by Jones et al*.*^26^. The annual heat sum for the flowering year was calculated from the first day of summer preceding the assessment year.

### Statistical analysis

The floral traits (start, end, duration of flowering and the number of reproductive flushes), biomass and foliar cineole of each tree were analysed using a series of mixed linear models in SAS 9.4^48^:

2a $$ {\text{y}}_{ijklmn} = {\text{r}}_{i} + {\text{c}}_{j} \left( {{\text{r}}_{i} } \right) + {\text{b}}_{k} \left( {{\text{r}}_{i} } \right) + {\text{p}}_{l} + {\text{f}}_{m} \left( {{\text{p}}_{l} } \right) + {\text{y}}_{n} + {\text{p}}_{l} .{\text{y}}_{n} + {\text{y}}_{n} .{\text{f}}_{m} \left( {{\text{p}}_{l} } \right) + {\text{e}}_{ijklmn} $$

2b $$ {\text{y}}_{ijklm} = {\text{r}}_{i} + {\text{c}}_{j} \left( {{\text{r}}_{i} } \right) + {\text{b}}_{k} \left( {{\text{r}}_{i} } \right) + {\text{p}}_{l} + {\text{f}}_{m} \left( {{\text{p}}_{l} } \right) + {\text{e}}_{ijklm} $$

where y_*ijklmn*_* and* y_*ijklm*_ are the floral, biomass and cineole traits, r_*i*_ is the random replicate effect, c_*j*_(r_*i*_) is the random effect column_*j*_ nested within replicate_*i*_, b_*k*_(r_*i*_) is the random effect row_*k*_ nested within replicate_*i*_, p_*l*_ is the provenance_*l*_, f_*m*_(p_*l*_) is the family_*m*_ within the provenance_*l*_, y_*n*_ is the year_n_ and e_*ijklmn*_ and y_*ijklm*_ are the residual errors of the respective models. Interactions were tested between year and provenance (p_l_.y_n_), as well as between year and family nested within provenance (y_n_.f_m_(p_l_); however, the proportion of dual flush trees, cineole and biomass analyses excluded the year factor y_*n*_ from the model (Eq. 2b) because these data were not collected over multiple years. Given that the dataset was unbalanced due to mortality and thinning, replicate was treated as a random effect so that inter-block information could be recovered^49^. Biomass data was transformed using natural logarithms to conform to homogeneity assumptions. The proportion of trees that flowered and proportion of dual flush trees were tested with the same model following arcsine transformation. Tukey–Kramer tests were used to determine the differences in the least square means of key traits. Start of flowering week was standardised to compare the 2012 and 2014 assessments. At the first assessment in 2012, 9% of the trees were flowering, and the 2014 data were truncated to match the 9% flowering from the 2012 assessment.

To estimate heritability, the data were analysed with ASReml Version 4.1^50^ using a linear mixed model (Eq. 2a). To estimate univariate heritability in flowering traits from 2012 and 2014, Eq. (2b) was used but family within the provenance was a random effect as required to calculate variance for heritability estimates^51^. The heritability analysis for number of flushes and propensity to flower in each year used a binomial model with a logit link. Genetic correlations were estimated from bivariate analysis of traits. Insignificant random effects were removed if they introduced instability to the model and log-likelihood was used to determine the model of best fit.

Eucalypts have a mixed breeding system in open-pollinated seed orchards and self-fertilisation is common^19^. Based on the outcome of the Sampson and Byrne^38^ study of a third closely related subspecies in the loxophleba group, *E*. *loxophleba* subsp. *supralaevis*, it is assumed that E_liss_ and E_grat_ have a mixed mating system. Griffin and Cotterill^20^ suggested using a coefficient of relationship of ρ = 1/1.25 to compensate for mixed mating and a selfing percentage of 30% for seed sourced from wild populations of *E. regnans*. This approach has been assessed and confirmed appropriate for correcting heritability estimates in an open-pollinated eucalypt progeny trial^52^ and subsequently applied to a series of E_liss_ progeny trials^18^.

Narrow-sense heritability was calculated from variance components of the individual traits using the formula:

3 $$ {\hat{\text{h}}}^{2} = \frac{{\upsigma _{a}^{2} }}{{\left( {\upsigma _{a}^{2} { } +\upsigma _{\upeta }^{2} } \right)}} $$

where $\hat{h}^{2}$ is the narrow sense heritability, σ^2^_*a*_ is the additive genetic variance and σ^2^_*ƞ*_ residual error component of variance.

Genetic and phenotypic correlations were calculated between all combinations of flowering traits (start, duration and end), dry biomass weight and foliar cineole concentration using the formula:

4 $$ {\text{r}} = \frac{{\upsigma _{xy}^{ } }}{{\sqrt {\upsigma _{x}^{2} *\upsigma _{y}^{2} } }} $$

where r is either *r*_*g*_, the genetic correlation, or *r*_*p*_, the phenotypic correlation. σ_*xy*_ is the additive genetic covariance for the genetic correlations and σ^2^_*x*_ and σ^2^_*y*_ are the additive genetic variances of the two traits. For the phenotypic correlations, σ_*xy*_ is the phenotypic covariance and σ^2^_*x*_ and σ^2^_*y*_ are the phenotypic variances for the traits.

## Results

### General flowering observations

**Figure 2:**
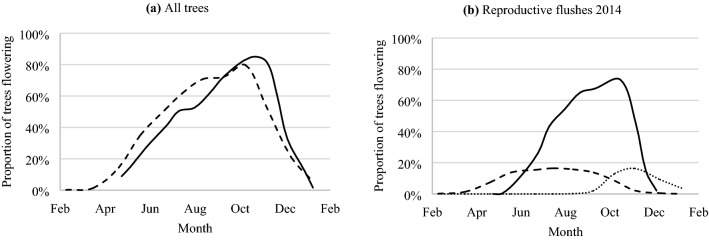
Proportion and timing of all trees flowering in the *Eucalyptus loxophleba* seed orchard at monthly intervals: (**a**) across all families and provenances in 2012 (solid line) and 2014 (dashed line); and (**b**) of single flush (black solid line) and the first (dashed line) and second (dotted line) flush of dual flush trees. Panels (**a**) and (**b**) were generated using Microsoft Excel, Version 2016. Microsoft Corporation, 2016 https://www.microsoft.com/en-au/download/office.aspx.

Of the population of 1142 trees, 87% flowered in each year (Table 2, Fig. 2a). The reproductive activity differed between the subspecies with E_grat_ from the two south provenances having the lowest (79–82%) flowering in both years (Table 2). E_liss_ showed consistent reproductive activity: 91% and 93% from the eastern and western provenances, respectively. From the monthly observations, the population commenced flowering in late summer to early autumn, with peak flowering in spring (Fig. 2a). Flowering was about 2 weeks earlier in 2014 and this trend continued for most of the assessment period. The earlier flowering in 2014 corresponded with a higher heat sum (4515 °C) compared to 2012 (4332 °C).

**Table 2:** Proportion of individuals flowering (%) for trees derived from each provenance for each assessment year and the proportion of trees with dual reproductive flushes (%) in the 2014 assessments.

Provenance	Region	% flower 2012	% flower 2014	% dual flush 2014
Dumbleyung	E_grat_ south	81.8	80.3	17.4
Lake Grace	E_grat_ south	82.8	78.4	21.2
Coolgardie	E_liss_ east	86.8	94.3	28.3
Goongarrie	E_liss_ east	84.6	94.9	5.1
Norseman	E_liss_ east	93.1	89.8	34.3
Narembeen	E_liss_ west	94.9	84.6	1.3
Southern Cross	E_liss_ west	83.3	91.1	4.4
Trayning	E_liss_ west	88.0	95.7	1.4
Westonia	E_liss_ west	92.0	92.0	8.0

Tracking of individual reproductive flushes in 2014 revealed that 16% of trees flowered on two separate occasions (Table 2). Two provenances from the E_liss_ east region had the highest proportion of dual flush trees followed by the two E_grat_ provenances. The trees that had dual reproductive phases exhibited two different flowering phenologies; the first flush of the dual flush trees had a long flowering duration compared to the second flush (Fig. 2b). In trees with a single flowering flush, the timing of flower initiation affected the duration of flowering. Trees that started flowering in late summer and autumn flowered for over 20 weeks, whereas a flowering phase that commenced in late winter and early spring flowered for under 12 weeks (Supplementary Table S1). In assessing the duration of flowering, flowering phases that commenced after week 38 were omitted because a high proportion had not concluded by the time the study was terminated.

### Effect of provenances on traits

**Figure 3:**
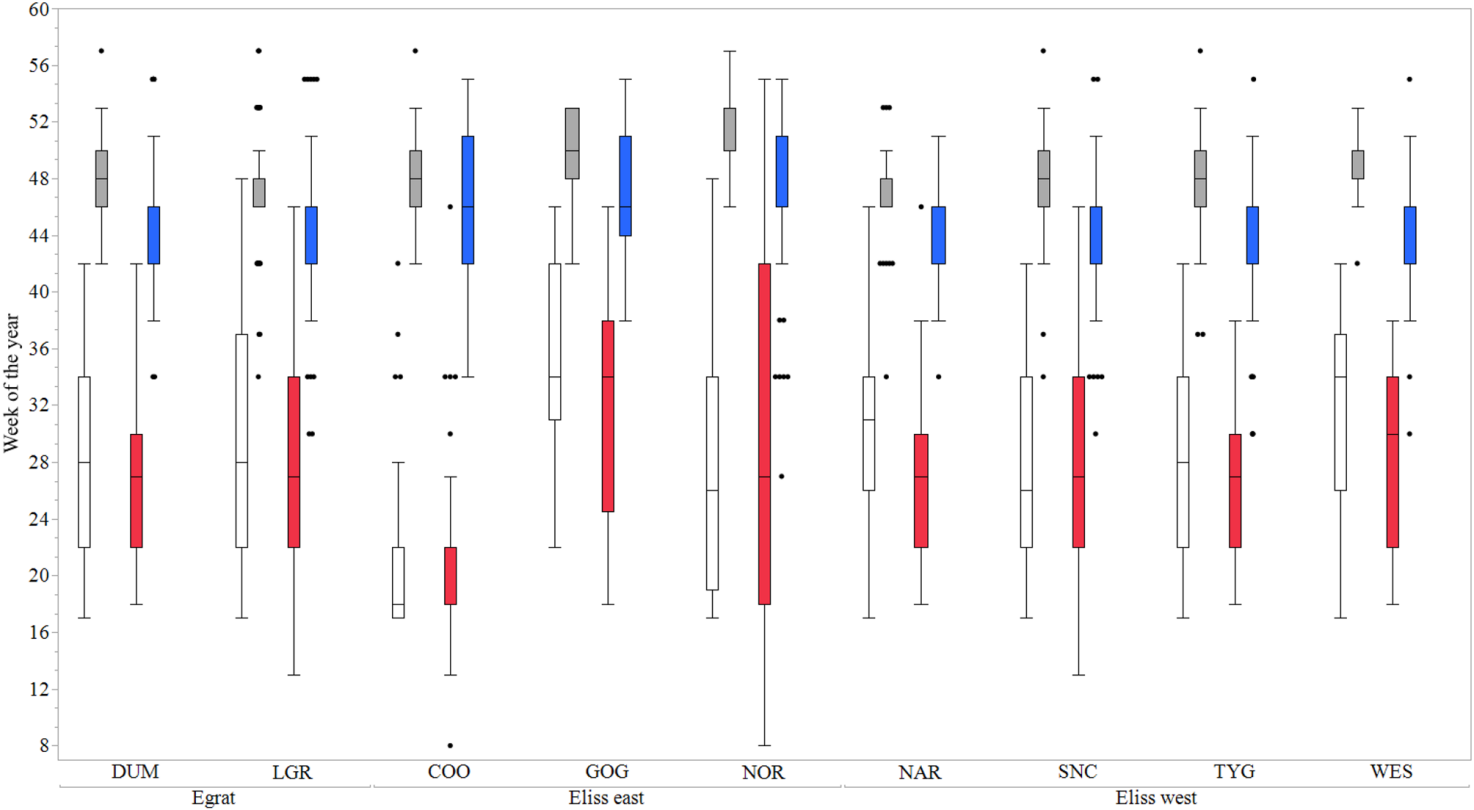
Start and end flowering times from 2012 and 2014 of *Eucalyptus loxophleba* provenances. Box plots represent variation in flowering times for 2012 start (no fill) and end (grey fill) and 2014 start (red fill) and end (blue fill). Flowering week is from the first week of January. Line in the middle of each box is the median provenance flowering time and edge of boxes 25th and 75th percentile. Dots represent outliers. Provenances are: *DUM* Dumbleyung, *LGR* Lake Grace, *COO* Coolgardie, *GOG* Goongarrie, *NOR* Norseman, *NAR* Narembeen, *SNC* Southern Cross, *TYG* Trayning, *WES* Westonia. Figure was generated using JMP, Version 14. SAS Institute Inc. 1989–2019 https://www.jmp.com/en_au/home.html.

Flowering start, end, duration, proportion of flowering, proportion of dual flush, foliar cineole content and biomass differed, significantly (*P* < 0.0001) between provenance and family within provenance and between family within provenance for start of flowering (*P* < 0.001) (Table 3). High levels of synchronicity in flowering events occurred within provenances, but there were substantial differences in the timing of flowering between provenances, as shown in (Fig. 3 and Table 4). For all the flowering traits assessed in this study, there were provenance by year interactions. Thus, a few provenances varied substantially between flowering years, with Dumbleyung, Southern Cross and Westonia finishing flowering four weeks later in 2014 compared to 2012. Also, the Norseman and Southern Cross provenances commenced flowering earlier in 2012 than in 2014 while all other provenances started flowering later. There were considerable provenance differences between flowering traits. The Coolgardie provenance commenced flowering earlier than the other provenances in both years, whereas the Goongarrie provenance started flowering later. All provenances finished flowering later in 2012 than 2014. Flowering duration was generally longer in 2014 than in 2012 with about a 4-week difference at Norseman and Southern Cross. Flowering was shortest in both years for E_liss_ provenances from Goongarrie. The other E_liss_ east provenances had some of the longest flowering duration. The E_grat_ provenances were less reproductively active across both years except when compared to Narembeen in 2014 and Southern Cross, Goongarrie and Coolgardie in 2012.

**Table 3:** Results of least square means analysis of flowering traits, cineole and biomass. *F*-values and degrees of freedom, in parentheses, for the fixed effects traits (start, end and duration of flowering, proportion flowering, proportion dual flush, foliar cineole concentration and pre- and post-thinning biomass estimates) and the *Z*-values for the random effects (replicate, column nested within replicate and row nested within replicate) at the *Eucalyptus loxophleba* seed orchard.

Effect	Flowering			Proportion		Foliar cineole	ln (dry biomass)	
	Start	End	Duration	Flowering	Dual flush		Pre-thinned	Post-thinned
Fixed effects	*F* (degree of freedom) or *Z* value	*F* (degree of freedom) or *Z* value	*F* (degree of freedom) or *Z* value	*F* (degree of freedom) or *Z* value	*F* (degree of freedom) or *Z* value	*F* (degree of freedom) or *Z* value	*F* (degree of freedom) or *Z* value	*F* (degree of freedom) or *Z* value
Year	11.1 (1)**	353.0 (1)***	34.2 (1)***	0.52 (1)				
Prov	17.2 (8)***	57.4 (8)***	26.7 (8)***	7.38 (8)***	20.98 (8)***	104.6 (8)***	28.6 (8)***	11.2 (8)***
Family(Prov)	3.1 (8)**	12.1 (51)***	4.4 (51)***	2.63 (51)***	4.29 (51)***	22.1 (51)***	4.1 (51)***	3.3 (51)***
Year*Prov	7.8 (51)***	3.6 (8)**	2.2 (8)*	2.51 (8)*				
Year*Family(Prov)	1.0 (51)	1.9 (51)**	1.1 (51)	1.05 (51)				
**Random effects**								
Rep	0.0	1.7*	0.0	2.0*	0.0	2.3*	2.7**	0.6
Col(Rep)	2.2*	2.5**	1.9**	1.9**	0.0	2.0	4.6***	4.0***
Row(Rep)	4.0***	2.3**	2.9***	0.0	1.0	2.4***	4.5***	2.9**
Residual	27.9***	28.0***	41.6***	32.1***	21.4***	49.3***	50.1***	20.2***

Significance test results for fixed and random effects are denoted as: *P < 0.05; **P < 0.001; ***P < 0.0001.

**Table 4:** Least square means of flowering traits for provenance by year with the standard error (in parentheses) at the *Eucalyptus loxophleba* seed orchard.

Region	Prov	Year	Start flowering		End flowering		Duration flowering		Proportion flowering	
			Estimate (SE)	Sig	Estimate (SE)	Sig	Estimate (SE)	Sig	Estimate (SE)	Sig
E_grat_	DUM	2012	28.4 (0.67)	BCD	47.8 (0.47)	BCD	27.5 (1.00)	A	1.29 (0.05)	CD
E_grat_	DUM	2014	26.9 (0.68)	CD	45.7 (0.45)	DE	24.7 (0.96)	ABC	1.26 (0.05)	D
E_grat_	LGR	2012	29.2 (0.48)	BC	48.1 (0.32)	BC	20.7 (0.66)	CDEF	1.30 (0.05)	CD
E_grat_	LGR	2014	27.6 (0.50)	CD	44.0 (0.32)	E	18.1 (0.67)	EFG	1.23 (0.05)	D
E_liss_ east	COO	2012	21.4 (1.02)	E	49.3 (0.55)	AB	16.0 (1.18)	G	1.36 (0.08)	ABCD
E_liss_ east	COO	2014	21.2 (0.97)	E	47.4 (0.52)	BCD	16.2 (1.11)	G	1.48 (0.08)	AB
E_liss_ east	GOG	2012	34.4 (1.21)	A	47.3 (0.24)	CD	19.0 (0.48)	EFG	1.34 (0.09)	ABCD
E_liss_ east	GOG	2014	32.2 (1.13)	AB	44.4 (0.25)	E	17.7 (0.49)	G	1.49 (0.09)	AB
E_liss_ east	NOR	2012	27.3 (0.50)	CD	47.3 (0.38)	BCD	17.9 (0.79)	EFG	1.47 (0.05)	AB
E_liss_ east	NOR	2014	28.8 (0.51)	BCD	44.3 (0.40)	E	17.5 (0.84)	EFG	1.42 (0.05)	AB
E_liss_ west	NAR	2012	30.4 (0.80)	ABC	50.6 (0.25)	A	24.4 (0.49)	AB	1.49 (0.07)	AB
E_liss_ west	NAR	2014	27.7 (0.86)	BCD	48.7 (0.25)	B	20.8 (0.50)	DE	1.32 (0.07)	BCD
E_liss_ west	SNC	2012	26.8 (0.80)	CD	48.0 (0.38)	BC	22.2 (0.78)	BCD	1.33 (0.06)	BCD
E_liss_ west	SNC	2014	27.3 (0.76)	CD	44.6 (0.36)	E	18.0 (0.75)	EFG	1.45 (0.06)	AB
E_liss_ west	TYG	2012	29.0 (0.52)	BCD	47.8 (0.26)	BC	19.8 (0.52)	DEFG	1.39 (0.05)	ABC
E_liss_ west	TYG	2014	26.8 (0.50)	D	44.3 (0.25)	E	18.4 (0.50)	FG	1.50 (0.05)	A
E_liss_ west	WES	2012	30.3 (1.03)	ABCD	48.5 (0.48)	BC	19.3 (1.01)	DEFG	1.46 (0.08)	AB
E_liss_ west	WES	2014	27.8 (1.03)	BCD	44.8 (0.48)	E	18.0 (1.01)	DEFG	1.46 (0.08)	AB

Proportion flowering was arcsine-transformed. Tukey’s tests were performed to determine the difference between provenances by year interaction, means that are similar have the same letter at α = 0.05 significance level (Sig). Provenances are: *DUM* Dumbleyung, *LGR* Lake Grace, *COO* Coolgardie, *GOG* Goongarrie, *NOR* Norseman, *NAR* Narembeen, *SNC* Southern Cross, *TYG* Trayning, *WES* Westonia.

Two of the E_liss_ east provenances, Norseman and Coolgardie, had the highest proportion of dual flush trees followed by the two E_grat_ provenances (Tables 2, 5). All of the E_liss_ west provenances had a consistently low (1–8%) percentage of dual flush trees. The average foliar cineole concentrations for each provenance ranged from 1.6% in Southern Cross to 2.7% of green leaf weight in Coolgardie (Table 5). On average, the E_liss_ east provenances tended to have higher cineole concentration, while the E_liss_ west region contained the two worst performing provenances. There was consistency in the biomass of different provenances between first and second measurement (Table 5). For the pre- and post-thinning growth, both E_grat_ provenances were ranked among the top performers along with E_liss_ from Goongarrie. Coolgardie and Westonia were the poorest performers in both assessments. On average, E_liss_ east and E_liss_ west were similar in their biomass ranking.

**Table 5:** Least square means for proportion dual flush (arcsine transform of proportion that flowered twice), foliar cineole content (% green weight) and biomass estimates of orchard pre-thinned (aged 5) and post-thinned (aged 15) at the *Eucalyptus loxophleba* seed orchard with the standard error in brackets.

Region	Prov	Proportion dual flush		Cineole		Log dry biomass pre-thinning		Log dry biomass post-thinning	
		Estimate (SE)	Sig	Estimate (SE)	Sig	Estimate (SE)	Sig	Estimate (SE)	Sig
E_grat_	DUM	0.32 (0.05)	BC	2.1 (0.04)	E	1.9 (0.05)	A	3.4 (0.06)	A
E_grat_	LGR	0.39 (0.04)	B	2.5 (0.03)	C	1.9 (0.05)	A	3.4 (0.05)	A
E_liss_ east	COO	0.44 (0.08)	AB	2.7 (0.05)	A	1.5 (0.06)	E	2.9 (0.09)	C
E_liss_ east	GOG	0.09 (0.09)	D	2.3 (0.06)	CDE	1.8 (0.06)	AB	3.5 (0.10)	A
E_liss_ east	NOR	0.60 (0.04)	A	2.5 (0.03)	BC	1.7 (0.05)	BC	3.1 (0.05)	BC
E_liss_ west	NAR	0.01 (0.07)	D	2.6 (0.05)	AB	1.8 (0.06)	BC	3.2 (0.07)	AB
E_liss_ west	SNC	0.08 (0.06)	D	1.6 (0.04)	F	1.6 (0.05)	DE	3.2 (0.07)	AB
E_liss_ west	TYG	0.02 (0.04)	D	2.3 (0.03)	D	1.7 (0.05)	CD	3.1 (0.05)	BC
E_liss_ west	WES	0.15 (0.08)	CD	1.8 (0.05)	F	1.5 (0.06)	DE	3.0 (0.09)	BC

Tukey’s tests were performed to test the difference between provenances, means that are similar have the same letter at α = 0.05 significance level (Sig). Provenances are: *DUM* Dumbleyung, *LGR* Lake Grace, *COO* Coolgardie, *GOG* Goongarrie, *NOR* Norseman, *NAR* Narembeen, *SNC* Southern Cross, *TYG* Trayning, *WES* Westonia.

### Effect of families on traits

**Figure 4:**
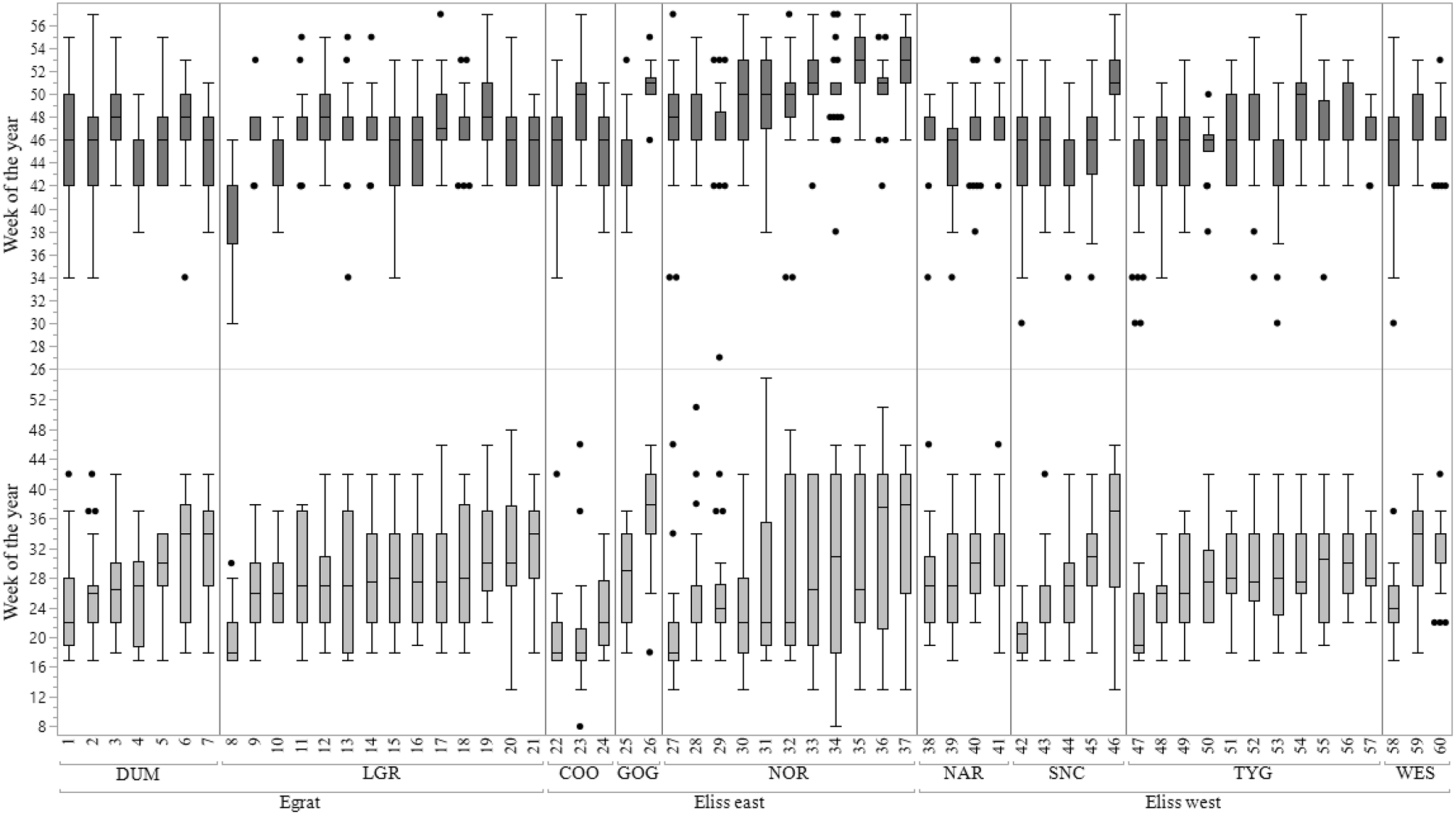
The start and end of flowering times (averaged over 2012 and 2014) of *Eucalyptus loxophleba lissophloia* (E_liss_) *E. loxophleba gratea* (E_grat_) families. Light grey box plots represent start flowering week and dark grey boxes plots represent end of flowering times. First week of January is week 1. Line in the middle of each box is the median provenance flowering time and edge of boxes 25th and 75th percentile. Dots represent outliers. *DUM* Dumbleyung, *LGR* Lake Grace, *COO* Coolgardie, *GOG* Goongarrie, *NOR* Norseman, *NAR* Narembeen, *SNC* Southern Cross, *TYG* Trayning, *WES* Westonia. Figure was generated using JMP, Version 14. SAS Institute Inc. 1989–2019 https://www.jmp.com/en_au/home.html.

There was substantial variation in flowering times within provenances. There was, for instance, about 3 months difference between the commencements of flowering among families derived from parents with Lake Grace, Norseman or Southern Cross provenance (Fig. 4). There was a spread of eight weeks in end of flowering within the individuals from the Lake Grace provenance and nearly 6 weeks range for Goongarrie and Norseman. There was also substantial overlap of flowering between most families although there are a few early or late families which were reproductively isolated from other families in the seed orchard. For instance, Family 8 (from Lake Grace) ended flowering in week 40 while Family 26 (from Goongarrie) started flowering in week 38 (details of the individual tree start and end of flowering times for families 8 and 26 is included in Supplementary Fig. S1). Generally, the peak flowering times of both of these families were out of phase with the peak flowering periods of the other families. The families that flowered the earliest (22, 8, 27 and 23) all started flowering on the twentieth week of the year whereas the last families to start flowering (46, 36, 37 and 26) started flowering 13–17 weeks later. A similar trend was observed with the end of flowering trait with the earliest families ending flowering in weeks 40–44 (8, 47, 10 and 53) and the latest end flowering families (26, 46, 37 and 35) ended after week 51. Least square means for all flowering traits of each family are detailed in Supplementary Table S2.

Table 6 shows substantial differences in the proportion of trees that flowered, proportion of trees with dual flushes, foliar cineole content and biomass from the families within provenances. The families within provenances that showed wide ranges in the proportion of trees that flowered were from Lake Grace (62–100%), Southern Cross (81–100%), and Dumbleyung (82–100%), while the rest had narrower ranges (90–100%). Similarly, there were families within provenances with considerable variation in dual flush proportions: in families from the Dumbleyung, Lake Grace, Coolgardie and Norseman provenances, the percentage of dual flush trees ranged from < 1 to 69%. For families from Goongarrie, there were no variation (both at 1%), while for the rest of the families within the remaining provenances, trees with dual flush were in the range of 0–7%.

**Table 6:** Least square means for foliar cineole content (% green weight), biomass estimates of pre-thinned (aged 5) and post-thinned (aged 15) orchard, arcsine transformed proportion of flowered and dual flush flowing with the standard error in brackets (SE).

Prov	Family	Foliar cineole (SE)	Log dry biomass pre-thinned (SE)	Log dry biomass post-thinned (SE)	Arcsine proportion flowered (SE)	Arcsine proportion dual flush (SE)
DUM	1	1.7 (0.09)	2.2 (0.08)	3.4 (0.14)	1.29 (0.09)	0.73 (0.14)
DUM	2	2.2 (0.10)	1.8 (0.09)	3.5 (0.14)	1.27 (0.09)	0.33 (0.14)
DUM	3	2.0 (0.08)	2.0 (0.08)	3.8 (0.14)	1.28 (0.09)	0.82 (0.13)
DUM	4	2.4 (0.09)	1.8 (0.09)	3.3 (0.15)	1.14 (0.10)	− 0.01 (0.16)
DUM	5	2.4 (0.08)	1.9 (0.08)	3.4 (0.14)	1.33 (0.09)	0.08 (0.13)
DUM	6	1.7 (0.08)	2.1 (0.08)	3.7 (0.14)	1.46 (0.09)	0.19 (0.13)
DUM	7	2.5 (0.08)	1.7 (0.08)	3.0 (0.14)	1.17 (0.08)	0.09 (0.13)
LGR	8	2.7 (0.09)	2.0 (0.09)	3.6 (0.16)	0.95 (0.09)	− 0.01 (0.18)
LGR	9	2.3 (0.08)	1.9 (0.08)	3.6 (0.14)	1.54 (0.09)	0.00 (0.12)
LGR	10	2.4 (0.08)	1.8 (0.08)	3.4 (0.14)	1.13 (0.08)	− 0.01 (0.15)
LGR	11	2.6 (0.08)	1.8 (0.08)	2.9 (0.14)	1.29 (0.09)	0.63 (0.14)
LGR	12	2.9 (0.08)	1.9 (0.08)	3.4 (0.14)	1.39 (0.09)	0.98 (0.13)
LGR	13	2.8 (0.08)	2.0 (0.08)	3.8 (0.14)	1.38 (0.09)	0.78 (0.13)
LGR	14	1.8 (0.09)	1.7 (0.08)	2.8 (0.15)	1.46 (0.09)	0.58 (0.13)
LGR	15	2.8 (0.08)	2.1 (0.08)	3.4 (0.14)	1.21 (0.09)	0.45 (0.14)
LGR	16	2.2 (0.08)	2.1 (0.08)	3.6 (0.14)	1.18 (0.08)	− 0.01 (0.13)
LGR	17	2.3 (0.08)	1.9 (0.08)	3.3 (0.14)	1.34 (0.08)	0.52 (0.12)
LGR	18	2.7 (0.08)	1.9 (0.08)	3.3 (0.14)	1.21 (0.09)	0.56 (0.14)
LGR	19	2.8 (0.09)	1.9 (0.09)	3.4 (0.14)	1.42 (0.08)	0.94 (0.12)
LGR	20	2.6 (0.08)	1.8 (0.08)	3.5 (0.14)	1.26 (0.09)	0.08 (0.12)
LGR	21	1.4 (0.08)	1.6 (0.08)	3.0 (0.15)	0.91 (0.09)	− 0.02 (0.19)
COO	22	3.2 (0.10)	1.4 (0.09)	2.7 (0.14)	1.39 (0.09)	0.37 (0.13)
COO	23	2.4 (0.08)	1.5 (0.08)	3.2 (0.14)	1.49 (0.09)	0.96 (0.12)
COO	24	2.5 (0.08)	1.5 (0.08)	2.7 (0.16)	1.38 (0.09)	0.00 (0.14)
GOG	25	2.5 (0.08)	1.9 (0.08)	3.2 (0.14)	1.25 (0.09)	0.09 (0.12)
GOG	26	2.2 (0.08)	1.8 (0.08)	3.9 (0.14)	1.58 (0.09)	0.09 (0.12)
NOR	27	2.7 (0.09)	1.7 (0.08)	3.1 (0.14)	1.45 (0.09)	0.78 (0.12)
NOR	28	2.6 (0.09)	1.8 (0.08)	3.6 (0.14)	1.38 (0.09)	0.09 (0.13)
NOR	29	2.5 (0.08)	1.9 (0.08)	3.5 (0.13)	1.22 (0.08)	0.22 (0.14)
NOR	30	2.5 (0.08)	1.8 (0.08)	3.2 (0.15)	1.53 (0.09)	0.83 (0.13)
NOR	31	2.4 (0.09)	1.8 (0.09)	3.1 (0.15)	1.45 (0.09)	0.97 (0.13)
NOR	32	2.2 (0.08)	1.7 (0.08)	2.9 (0.15)	1.56 (0.09)	0.52 (0.12)
NOR	33	2.4 (0.08)	1.7 (0.08)	3.0 (0.14)	1.49 (0.08)	0.83 (0.12)
NOR	34	2.6 (0.08)	1.4 (0.08)	2.6 (0.14)	1.33 (0.09)	0.78 (0.13)
NOR	35	2.6 (0.08)	1.7 (0.08)	2.9 (0.14)	1.49 (0.09)	0.60 (0.12)
NOR	36	2.5 (0.08)	1.6 (0.08)	3.2 (0.14)	1.50 (0.09)	0.41 (0.12)
NOR	37	2.4 (0.08)	1.8 (0.08)	3.1 (0.13)	1.46 (0.08)	0.52 (0.12)
NAR	38	2.6 (0.08)	1.7 (0.08)	3.1 (0.13)	1.36 (0.09)	− 0.01 (0.14)
NAR	39	2.7 (0.08)	1.8 (0.08)	3.3 (0.14)	1.52 (0.08)	0.07 (0.12)
NAR	40	2.5 (0.08)	1.8 (0.08)	3.1 (0.15)	1.35 (0.09)	0.00 (0.14)
NAR	41	2.5 (0.08)	1.8 (0.08)	3.3 (0.14)	1.41 (0.09)	− 0.01 (0.13)
SNC	42	1.5 (0.08)	1.7 (0.08)	3.2 (0.14)	1.40 (0.09)	0.09 (0.12)
SNC	43	2.3 (0.08)	1.8 (0.08)	3.4 (0.14)	1.43 (0.09)	0.00 (0.12)
SNC	44	1.5 (0.08)	1.7 (0.08)	3.1 (0.14)	1.12 (0.09)	0.11 (0.14)
SNC	45	1.9 (0.08)	1.6 (0.08)	3.5 (0.15)	1.40 (0.09)	0.00 (0.13)
SNC	46	0.9 (0.08)	1.3 (0.08)	2.8 (0.16)	1.59 (0.10)	0.22 (0.14)
TYG	47	3.1 (0.09)	1.6 (0.08)	2.9 (0.14)	1.45 (0.09)	0.00 (0.12)
TYG	48	2.0 (0.08)	1.7 (0.08)	3.0 (0.14)	1.58 (0.09)	0.08 (0.12)
TYG	49	2.1 (0.08)	1.8 (0.08)	3.4 (0.14)	1.44 (0.09)	0.00 (0.12)
TYG	50	2.1 (0.09)	1.7 (0.08)	3.1 (0.14)	1.56 (0.09)	− 0.01 (0.13)
TYG	51	2.0 (0.08)	1.5 (0.08)	3.0 (0.17)	1.51 (0.10)	− 0.01 (0.15)
TYG	52	2.6 (0.08)	1.6 (0.08)	3.3 (0.14)	1.42 (0.08)	0.07 (0.12)
TYG	53	2.5 (0.09)	1.7 (0.08)	3.0 (0.14)	1.42 (0.09)	0.00 (0.12)
TYG	54	2.3 (0.08)	1.9 (0.08)	3.1 (0.14)	1.34 (0.08)	0.08 (0.12)
TYG	55	2.2 (0.09)	1.6 (0.08)	3.3 (0.15)	1.40 (0.09)	− 0.01 (0.13)
TYG	56	2.3 (0.09)	1.6 (0.09)	3.0 (0.14)	1.27 (0.09)	0.00 (0.12)
TYG	57	2.5 (0.08)	1.7 (0.08)	3.2 (0.14)	1.49 (0.09)	− 0.01 (0.12)
WES	58	0.9 (0.09)	1.5 (0.08)	3.0 (0.16)	1.38 (0.10)	0.27 (0.15)
WES	59	2.6 (0.09)	1.5 (0.09)	3.0 (0.14)	1.46 (0.09)	0.19 (0.13)
WES	60	1.8 (0.08)	1.6 (0.08)	3.0 (0.14)	1.54 (0.09)	0.00 (0.12)

*DUM* Dumbleyung, *LGR* Lake Grace, *COO* Coolgardie, *GOG* Goongarrie, *NOR* Norseman, *NAR* Narembeen, *SNC* Southern Cross, *TYG* Trayning, *WES* Westonia.

There were two families (46 and 58) that averaged foliar cineole under 0.9% of green leaf weight whereas the top performing families had cineole concentration exceeding 3.0%. Several provenances have tight ranges (little variation among families) while other provenances show wide ranges between families. For example, families within the Narembeen provenance show little variation (2.5–2.7%), whereas those within Westonia are highly variable (0.9–2.6%). Likewise, there is more variation in biomass estimates across both measurements within the Lake Grace provenance compared to Westonia. There was nearly double the average back-transformed biomass from the high yielding families in the pre-thinned assessment (1, 6, 15 and 16 vs 59, 34, 22 and 46) and this difference was more pronounced after thinning (26, 3, 13 and 6 vs 46, 24, 22 and 34).

### Heritability, genetic and phenotypic correlations

Narrow-sense heritability ranged from high for end of flowering ($\hat{h}^{2}$ = 0.66–0.75), moderate for start flowering traits ($\hat{h}^{2}$ = 0.45 ± 0.10) and foliar cineole content ($\hat{h}^{2}$ = 0.53 ± 0.09) and low for duration of flowering and both biomass estimates ($\hat{h}^{2}$ = 0.10–0.33) (Table 7). Narrow-sense heritability for dual flush flowering was ($\hat{h}^{2}$ = 0.61 ± 0.16), propensity to flower in 2012 ($\hat{h}^{2}$ = 0.19 ± 0.10) and 2014 ($\hat{h}^{2}$ = 0.24 ± 0.11). There were strong genetic correlations between the same flowering traits from different years (*r*_*g*_ = 0.84–0.96) but the phenotypic correlations were lower (*r*_*p*_ = 0.40–0.55). Genetic correlations were also high between most of the key flowering traits across years except for end of flowering and duration of flowering which generally had high standard errors. The genetic correlations between the start and end of flowering were all positive and above 0.62. The magnitudes of the genetic correlations between start and duration of flowering were also high although these correlations were negative. Phenotypic correlations were generally lower than genetic correlations, but they had lower standard errors. There were high negative phenotypic correlations between start of flowering 2012 and duration of flowering in 2012 (*r*_*p*_ = − 0.93) and in 2014 (*r*_*p*_ = − 0.81).

**Table 7:** Heritability, genetic and phenotypic correlations for *Eucalyptus loxophleba* seed orchard.

	Genetic correlations								
	Start flower 12	Start flower 14	End flower 12	End flower 14	Duration flower12	Duration flower14	Cineole	Ln (DBM) pre-thinned	Ln(DBM) post-thinned
**Phenotypic correlations**									
Start flower 12	**0.446 ± 0.097**	0.888 ± 0.057	0.654 ± 0.109	0.739 ± 0.093	− 0.905 ± 0.034	− 0.592 ± 0.156	− 0.099 ± 0.162	− 0.291 ± 0.173	− 0.118 ± 0.190
Start flower 14	*0.549* ± *0.028*	**0.447 ± 0.097**	0.628 ± 0.115	0.797 ± 0.078	− 0.807 ± 0.086	− 0.690 ± 0.109	− 0.276 ± 0.154	− 0.456 ± 0.157	− 0.158 ± 0.188
End flower 12	*0.335* ± *0.041*	*0.336* ± *0.043*	**0.655 ± 0.117**	0.963 ± 0.030	− 0.258 ± 0.177	0.154 ± 0.206	− 0.209 ± 0.150	− 0.211 ± 0.171	− 0.043 ± 0.184
End flower 14	*0.309* ± *0.042*	*0.395* ± *0.038*	*0.540* ± *0.035*	**0.746 ± 0.123**	− 0.396 ± 0.159	− 0.118 ± 0.205	− 0.244 ± 0.147	− 0.227 ± 0.168	− 0.038 ± 0.181
Duration flower12	− *0.931* ± *0.006*	− *0.476* ± *0.031*	*0.017* ± *0.046*	− *0.136* ± *0.044*	**0.328 ± 0.083**	0.843 ± 0.114	− 0.001 ± 0.173	0.256 ± 0.185	0.126 ± 0.200
Duration flower14	− *0.377* ± *0.034*	− *0.814* ± *0.015*	− *0.014* ± *0.044*	*0.191* ± *0.040*	*0.400* ± *0.034*	**0.186 ± 0.063**	0.127 ± 0.194	0.493 ± 0.185	0.240 ± 0.217
Cineole	− *0.003* ± *0.048*	− *0.061* ± *0.048*	*0.226* ± *0.034*	*0.171* ± *0.033*	− *0.032* ± *0.047*	*0.030* ± *0.046*	**0.526 ± 0.087**	0.209 ± 0.160	0.180 ± 0.167
Ln (DBM) pre-thinned	− *0.114* ± *0.036*	− *0.054* ± *0.037*	*0.036* ± *0.037*	*0.125* ± *0.031*	*0.131* ± *0.037*	*0.034* ± *0.038*	− *0.015* ± *0.02*	**0.096 ± 0.025**	0.763 ± 0.091
Ln (DBM) post-thinned	− *0.139* ± *0.042*	− *0.123* ± *0.041*	*0.037* ± *0.045*	− *0.048* ± *0.044*	*0.164* ± *0.041*	*0.080* ± *0.041*	*0.047* ± *0.045*	*0.678* ± *0.017*	**0.288 ± 0.075**

Narrow-sense heritability (± standard errors) of each trait is on the diagonal (in bold), above the diagonal is genetic correlation (± standard errors) and below the diagonal is phenotypic correlations (± standard errors) in italics. Traits are start flowering week in 2012 and 2014, end of flowering week in 2012 and 2014, duration of flowering in 2012 and 2014, foliar cineole concentration by weight, ln (dry biomass) of the pre-thinned orchard as measured in 2004 and ln (dry biomass) of post-thinned seed orchard as measured in 2014.

The correlations were of similar magnitude to the standard errors for many of the biomass and cineole estimates, except between the two biomass estimates. There were however, moderate genetic correlations between flowering duration in 2014 and pre-thinned biomass, and weak genetic correlations between start and end of flowering time in 2014 and foliar cineole concentration. In contrast, there was a weak positive phenotypic correlation between cineole and of end of flowering in 2012 and 2014. There were also weak negative phenotypic correlations between post-thinned biomass and start of flowering. There was a moderate negative genetic correlation between pre-thinned biomass and start of flowering in the 2014 assessment and a corresponding positive weak phenotypic correlation end of flowering for 2014.

## Discussion

Understanding flowering phenology is critical in seed orchard design, as synchronicity of flowering amongst parents is key to maximising outcrossing, genetic quality of the seed, and its ability to deliver traits of commercial interest. The results from this study displayed large variation in flowering phenology within and between subspecies, provenances and families. With the exception of a few families, there was a high level of synchronicity of flowering within the seed orchard but substantial differences between provenances. However, caution should be used when directly comparing the provenance-level data as there are different numbers of families in each provenance; for example, Coolgardie, Goongarrie and Westonia were poorly represented with a small number of families.

The large range of commencement and end of flowering times within provenances may in part be reflected by the percentage of dual-flush trees in each provenance. In most cases, dual flush trees started flowering earlier (i.e. the first flush) in the year than their single flush contemporaries, resulting in greater flowering duration. These were the most of the out-of-phase trees in the orchard. We estimate that only a small proportion (< 4%) of the total buds in the orchard flowered out of phase with the single flush trees. Early dual flush trees which were active before the main flush of the orchard will have a greatly reduced opportunity to outcross with other trees in the seed orchard.

After the E_liss_ eastern provenances the southern E_grat_ provenances had the second highest proportion of dual flush trees. There is no evidence that the two regions exhibiting high rates of dual flush flowering is due to their relatedness. Investigation on the chloroplast DNA has revealed that E_grat_ is more closely related to the neighbouring E_liss_ western and *E. loxophleba* subsp. *loxophleba* than to E_liss_ eastern provenances^53^. However, the high heritability of the dual flush trait ($\hat{h}^{2}$ = 0.61 ± 0.15) suggests that selections could be made to reduce its prevalence in breeding populations. A large percentage of these dual flush trees were E_grat_, and based on this alone, it may be appropriate to separate E_grat_ into a separate breeding population. The taxonomic split between E_liss_ and E_grat_ is contentious^54,55^, however, a breeding population comprised of E_grat_ alone would allow more out-crossing of these dual flush trees especially at the start and end of the annual reproductive cycles. Considering E_liss_ has been shown to have weak reproductive barriers^38–42^, it would allow the subspecies to be planted in their natural range thereby minimising genetic ‘pollution’ of other subspecies.

The dual flush E_liss_ trees, most of which originate from eastern provenances, flowered out of phase with the single flush E_liss_. Many of these families also performed poorly in biomass assessments. For example, the Coolgardie provenance (families 22, 23 and 24) ranked last for biomass in the two measurements of this trial, flowered considerably earlier than any other family and nearly 30% of the trees flowered twice. Family 22 had the highest foliar cineole concentration, but ranked second and third last for biomass and was the earliest family to flower. Some families in the Norseman provenance, in contrast, included seven of the latest ten flowering families with a high proportion of dual flush trees and below average biomass. High cineole families may be kept in the seed orchard to maintain cineole levels, but seed should not be collected from these families for biomass plantings and they should be carefully considered before inclusion in future breeding programmes. Families with a high proportion of dual flush trees, low cineole and biomass rank, could be removed from the breeding populations. The other E_liss_ eastern provenance, Goongarrie, consisted of two families, both with a low proportion of dual flush trees. Family 26 was the fourth latest to flower, but this family ranked highest in biomass in the second assessment and family 25 flowered during peak time and ranked well for both biomass and cineole. Thus, although E_liss_ east provenances were on average poor performers in the seed orchard, some families from the eastern provenances may be candidates for next-generation seed or clonal orchards.

Mazanec et al*.*^18^ reports on the variation of biomass from different provenances from three large E_liss_ progeny trials across southern Australia. Each trial consisted of nine provenances with at least 13 families. It was found that the progeny from Norseman ranked last for biomass at two of the three trials and the progeny from Cardunia Rocks, the other eastern provenance, performed average or below at the three trials. These trials were measured at age three and gives further support for the poor biomass performance from the E_liss_ eastern range. However, short-duration studies can at times fail to indicate longer-term performance and further measurements of these trial should reveal more about the top biomass producing E_liss_ provenances.

This study shows that, with the exception of a few families, there is a considerable proportion of trees flowering in the seed orchard throughout most months the year. However, the amount of reproductive activity is uneven; the rate of anthesis is much more pronounced in late winter and spring when it is common for 30–50% of buds to have commenced flowering in a 1-month period. This contrasts with other studies of southern *Eucalyptus* species. For example, warmer temperatures are known to trigger key developmental stages in *E. nitens* including bud initiation and growth^56^ and heat-sum has been found to be the main driver of anthesis time in *E. globulus* and *E. nitens*^26,57^. With only 2 years of data, the effect that heat sum had on the timing of anthesis for *E. loxophleba* is impossible to determine. The year of greater heat did correspond with earlier flowering and shorter flowering duration, both expected outcomes in the heat sum model. However, E_liss_ and E_grat,_ in contrast to *E. nitens* and *E. globulus*, are adapted to arid conditions.

Moisture availability and rainfall events have been identified as important factors affecting anthesis for plants in arid environments^58–62^ and that is a likely cause of slow reproductive development of E_liss_ and E_grat_ during late summer and autumn. Furthermore, Friedel et al*.*^59^ demonstrated that soil moisture was a predictor of flowering for 46 arid-zone species although flowering events lagged between 1 and 9 months after rainfall. This is consistent with the flowering of E_liss_ and E_grat_ in this study where there were no significant rainfall events in 2012 until May and in 2014 until April. E_liss_ and E_grat_ are well adapted to drought and appear to exhibit drought-induced dormancy of the crown.

The plastic nature of the species recorded in the arid zone of Australia by Friedel et al*.*^59^, Davies^61^ and others suggest that eastern provenances of E_liss_ may be more strongly adapted to rainfall-induced growth and reproduction than western provenances. The multiple reproductive events observed in this trial may be a function of that adaptation. It is possible that trees from the low rainfall eastern provenances were responding to the higher rainfall experienced near Lake Toolibin. However, the two most southerly provenances (E_grat_) also had a high percentage (20%) of dual flushes trees and are the two closest provenances to Lake Toolibin. E_grat_ was less reproductively active, suggesting that these provenances did not benefit from the slightly higher rainfall of the study site when compared to their natural range. In contrast, western and eastern E_liss_ provenances displayed high levels of reproductive activity at a site with higher rainfall and lower evaporation compared to their natural distribution. There was however, no trend in northern provenances to flower earlier than the more southerly E_grat_ provenances, a trend recorded for *E. marginata*^63^ and for *Corymbia citriodora* subsp. *variegata* and *C. maculata*^27^. However, the opposite trend has been observed in *E. globulus* where Victorian provenances flowered later than the more southerly eastern-coast Tasmanian provenances^26^.

Biomass generally has a lower heritability than either flowering traits or cineole production and this was observed in this trial. The pre-thinned biomass heritability was low ($\hat{h}^{2}$ = 0.10 ± 0.03) but increased after thinning to 0.29 ± 0.08 suggesting that estimates of additive genetic variance for this trait were biased upwards as a result. The difference between the two estimates suggests that some degree of bias was introduced as a result of selective thinning. We have no way of knowing if the bias extends to the flowering traits; and therefore, some caution should be used in interpretation of these results. However, Mazanec et al*.*^18^ found narrow-sense heritably at three E_liss_ progeny trials of 0.19 ± 0.05, 0.13 ± 0.04 and 0.25 ± 0.05 at Monarto (South Australia), Condobolin (New South Wales) and Brookton (Western Australia) respectively. Stem diameter, an accurate estimator of biomass, has been found to be moderately heritable in other eucalyptus species including *E. nitens *$\hat{h}^{2}$ = 0.18–0.19^64,65^, and *E. cladocalyx *$\hat{h}^{2}$ = 0.14^66^ and $\hat{h}^{2}$ = 0.30^67^.

Heritability of flowering traits in this study were under moderate to strong genetic control. These results are consistent with results from other studies in eucalypts. Jones et al*.*^26^ reported high broad-sense heritability for peak anthesis time ($\hat{H}^{2}$ = 0.78) in an *E. globulus* clonal seed orchard but found weak heritability for duration of flowering ($\hat{H}^{2}$ = 0.09) with the low heritability attributed to the correlation between duration of flowering and flower abundance^68^. For the same species, Gore and Potts^25^ found narrow-sense heritability over 0.64 for start, peak and end of flowering after a single year of assessment. Flowering intensity of *E. cladocalyx* has been recorded as $\hat{h}^{2}$ = 0.48^69^ and $\hat{h}^{2}$ = 0.52^66^. The number of reproductive flushes was under strong genetic control ($\hat{h}^{2}$ = 0.61 ± 0.16) but the propensity to flower in 2012 and 2014 were quite low $\hat{h}^{2}$ = 0.19 ± 0.10 and $\hat{h}^{2}$ = 0.24 ± 0.11 respectively, although this is much higher than the $\hat{h}^{2}$ = 0.06 ± 0.05 reported for *E. globulus*^26^.

Foliar cineole concentration was observed to be under strong genetic control ($\hat{h}^{2}$ = 0.53 ± 0.09). Mazanec et al*.*^18^ found similar narrow-sense heritability at an E_liss_ progeny trial in Brookton, Western Australia, of over 1700 trees from 126 families ($\hat{h}^{2}$ = 0.53 ± 0.07) and from an E_grat_ progeny trial of 90 families of ($\hat{h}^{2}$ = 0.50 ± 0.08)^70^. Similar heritability was found in *E. camaldulensis*^29^. Heritability as high as $\hat{h}^{2}$ = 0.83 was found in a small *E. kochii* seed orchard^71^. In the current study, the parent trees had been tested for foliar cineole concentration prior to selection with seed only sourced from elite individuals (foliar cineole concentration > 2.5%) so selection was biased to high cineole individuals. This is because at that time, in the genetic selection of this species, cineole was considered likely to be the major product for commercial planting of these species^8,11^. The two more recent E_liss_ and E_grat_ progeny trials mentioned above were not subject to pre-selection for foliar cineole but the heritability results were similar suggesting that pre-selection for high cineole did not influence estimates of additive variance in this trial.

The strong genetic and phenotypic correlations between duration and start of flowering have been found in other species, for instance, *Lythrum salicaria* (*r*_*p*_ = − 0.92)^72^. The correlations for E_liss_ are much higher when comparing within years than across years and may be attributable to the annual variation in dual flush flowering which exhibit longer duration of flowering. It is unknown if the trees that flowered twice in 2014 also did so in 2012 and although the heritability for this trait is high, another assessment following individual reproductive flushes would be useful. Furthermore, assessment of the performance of progeny from the first-and second-flush of the dual flush trees and single flush trees could indicate the degree of inbreeding or selfing.

Genetic correlations suggest that selection for high biomass and cineole may result in selection of early flowering trees. For example, if selection is biased toward biomass then the low genetic correlation for that trait with flowering time will allow greater freedom in selection of trees with later flowering times. The orchard was initially conservatively thinned, in most cases an individual from each family was retained from each replicate. Poor biomass producing E_liss_ east families with high levels of dual flush flowering could be eliminated from the orchard which would increase flowering synchronicity without negatively impact biomass production.

## Conclusion

This study has shown that timing of anthesis is strongly influenced by genetic factors. Genetically, there is a large amount of variation with broad-scale differences among provenances and families. Most flowering traits, along with cineole, were moderately to strongly heritable whereas biomass heritability was low. At the start and end of the annual flowering cycle, dual flush trees were reproductively isolated and because the trait is strongly heritable, this could result in greater flowering asymmetry in progeny collected from the dual flush trees. Dual flush flowering was evident in the E_grat_ provenances. For this reason and because of the potential spread of genetic material to native stands through pollen dispersal, E_grat_ should be treated as a separate breeding population for use within its natural range. The E_liss_ east provenances included families with the highest proportion of dual flush trees and the poorest biomass yield. These families should be eliminated from the general breeding programme. Due to the moderate to negligible genetic correlations between flowering traits, biomass and foliar cineole concentrations, selections should be based on biomass and cineole. Families with a high proportion of dual flush trees and otherwise desirable characteristics should be further studied to determine if this was a one-off event or if it is a recurring phenomenon. Opportunistic use of available soil moisture may be responsible for dual flush flowering in eastern provenances, however, the 2-week divergence in flowering time between assessment years may be driven by heat-sum.

## Supplementary information

Supplementary Information.

## Data Availability

The flowering key traits (start, end and duration of flowering), biomass estimates and foliar cineole data from this manuscript has not been achieved, but prior to publication it will be published on CSIRO data access portal https://data.csiro.au/dap/discoveryService with a DOI number.
