# Supplementary material for: Flowering phenology in a Eucalyptus loxophleba seed orchard, heritability and genetic correlation with biomass production and cineole: breeding strategy implications
Source: Sci Rep. 2020 Sep 17;10:15303. doi: 10.1038/s41598-020-72346-3 (PMC7499158; doi:10.1038/s41598-020-72346-3)
Supplement: Supplementary file 1 — Supplementary Information. [file 41598_2020_72346_MOESM1_ESM.pdf]

Supplementary Material

Flowering phenology in a *Eucalyptus loxophleba* seed orchard, heritability and genetic correlation with biomass production and cineole – breeding strategy implications

Beren Spencer[12]*, Richard Mazanec[2], Amir Abadi[1], Mark Gibberd[1] and Ayalsew Zerihun[1]

[1] School of Molecular and Life Science, Curtin University, GPO Box U1987, Perth, WA, Australia, 6845
[2] Department of Biodiversity, Conservation and Attractions, Kensington, WA, Australia, 6151

*Corresponding author. Email address: beren.spencer-@postgrad.curtin.edu.au. Phone: +61 426278882
Primary research manuscript

Table S1 – The duration of a flowering phase in the 2014 assessment determined by the month flowering commenced. Separate assessments were made for trees that underwent two reproductive events.

Month start flowering	No. trees flowering	Average flowering duration (weeks)
February	2	21.5
March	13	22.4
April	151	22.3
May	240	20.4
June	189	17.8
July	127	15.7
August	120	12.7
September	77	10.9

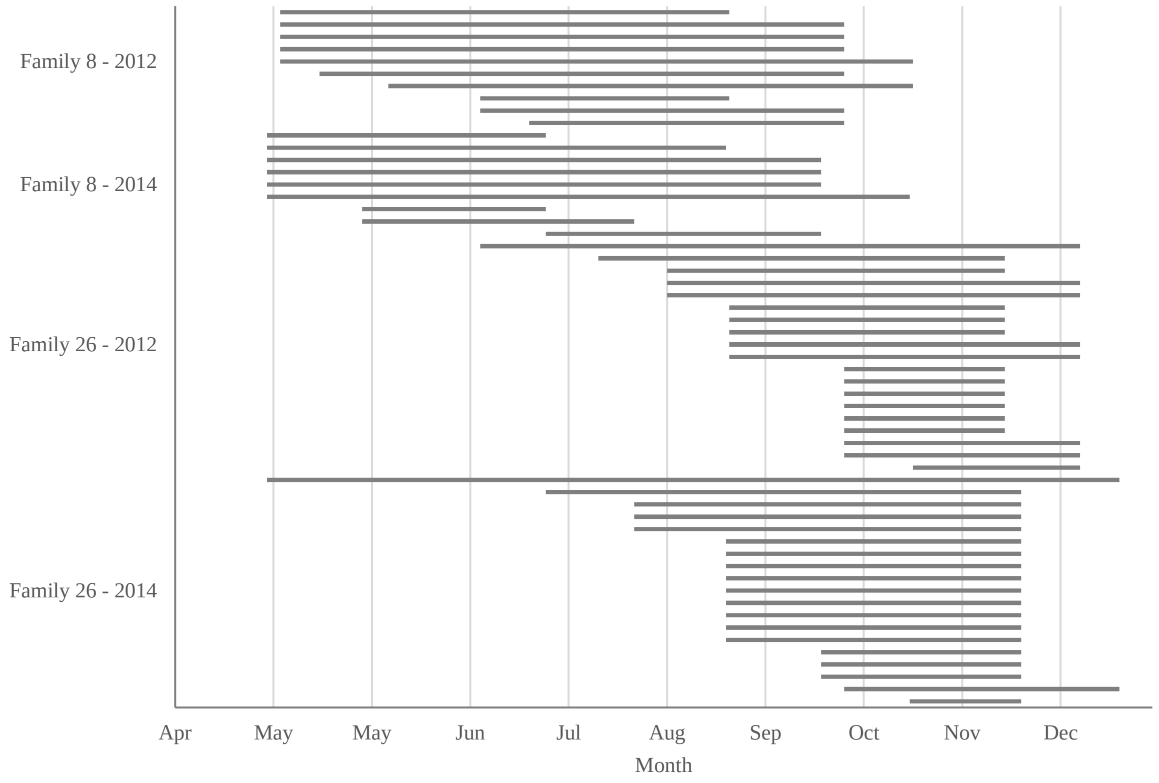

Fig. S1 Variation in flowering time for individual trees from two families (8 and 26) over two years (2012 and 2014). Family 8 was the earliest flowering family and Family 26 was the latest.

Table S2 – Least square means of each family for start flowering week, end flowering week and duration of flowering with the standard error in brackets (SE). Untransformed proportion or individuals flowering (%) in 2012 and 2014, and the proportion of trees that flowered twice (dual flowering) from each family. DUM = Dumbleyung, LGR = Lake Grace, COO = Coolgardie, GOG = Goongarrie, NOR = Norseman, NAR = Narembeen, SNC = Southern Cross, TYG = Trayning, WES = Westonia.

Provenance	Family	Start flowering week (SE)	End flowering week (SE)	Duration flowering (SE)	% flowered 2012	% flowered 2014	% dual flowering
DUM	1	24.3 (1.2)	46.4 (0.6)	23.2 (1.2)	84.2	78.9	36.8
DUM	2	26.0 (1.3)	46.1 (0.6)	21.2 (1.3)	83.3	77.8	16.7
DUM	3	26.0 (1.2)	48.0 (0.6)	23.3 (1.2)	75.0	85.0	45.0
DUM	4	26.2 (1.5)	44.2 (0.7)	19.0 (1.5)	73.3	73.3	0.0
DUM	5	28.9 (1.2)	45.5 (0.6)	17.5 (1.2)	85.0	85.0	5.0
DUM	6	30.9 (1.2)	46.8 (0.5)	17.1 (1.2)	100.0	84.2	10.5
DUM	7	31.8 (1.2)	45.1 (0.6)	14.5 (1.2)	71.4	76.2	4.8
LGR	8	20.4 (1.6)	40.2 (0.7)	21.1 (1.6)	62.5	56.3	0.0
LGR	9	26.3 (1.1)	45.9 (0.5)	20.6 (1.1)	95.0	100.0	0.0
LGR	10	26.7 (1.3)	43.7 (0.6)	18.1 (1.3)	81.0	61.9	0.0
LGR	11	27.3 (1.2)	46.5 (0.6)	20.3 (1.2)	84.2	78.9	31.6
LGR	12	27.4 (1.2)	47.4 (0.5)	21.0 (1.2)	95.0	80.0	50.0
LGR	13	28.2 (1.2)	46.4 (0.5)	19.3 (1.2)	95.0	80.0	40.0
LGR	14	28.2 (1.2)	46.5 (0.6)	19.4 (1.2)	94.1	94.1	35.3
LGR	15	29.3 (1.2)	45.5 (0.6)	17.0 (1.2)	85.0	70.0	20.0
LGR	16	29.4 (1.3)	45.4 (0.6)	17.0 (1.3)	65.0	85.0	0.0
LGR	17	29.6 (1.1)	47.5 (0.5)	18.9 (1.2)	85.7	85.7	28.6
LGR	18	30.7 (1.2)	46.9 (0.6)	17.1 (1.2)	85.0	70.0	25.0
LGR	19	31.0 (1.1)	48.3 (0.5)	18.3 (1.1)	90.9	90.9	54.5
LGR	20	31.5 (1.2)	46.1 (0.6)	15.6 (1.2)	70.0	90.0	5.0
LGR	21	32.9 (1.6)	45.4 (0.7)	13.5 (1.6)	64.7	47.1	0.0
COO	22	20.0 (1.2)	44.8 (0.6)	26.1 (1.2)	83.3	94.4	22.2
COO	23	20.8 (1.1)	49.4 (0.5)	29.7 (1.2)	94.7	94.7	57.9
COO	24	24.6 (1.3)	46.1 (0.6)	22.5 (1.3)	81.3	93.8	0.0
GOG	25	29.0 (1.2)	45.6 (0.6)	17.7 (1.2)	70.0	90.0	5.0
GOG	26	37.7 (1.1)	51.2 (0.5)	14.5 (1.1)	100.0	100.0	5.3
NOR	27	20.6 (1.1)	46.9 (0.5)	28.1 (1.1)	95.0	90.0	45.0
NOR	28	25.1 (1.2)	47.3 (0.5)	23.2 (1.2)	90.0	85.0	5.0
NOR	29	25.2 (1.2)	46.5 (0.6)	22.3 (1.2)	86.4	68.2	9.1
NOR	30	25.8 (1.2)	50.0 (0.5)	25.7 (1.2)	100.0	94.4	50.0
NOR	31	27.4 (1.2)	49.8 (0.6)	23.7 (1.2)	94.4	88.9	55.6
NOR	32	28.2 (1.1)	49.3 (0.5)	22.3 (1.2)	100.0	100.0	33.3
NOR	33	29.1 (1.1)	50.9 (0.5)	23.4 (1.1)	95.0	95.0	50.0
NOR	34	30.2 (1.2)	50.1 (0.6)	20.9 (1.2)	84.2	84.2	42.1
NOR	35	31.6 (1.1)	52.3 (0.5)	21.9 (1.1)	100.0	90.0	35.0
NOR	36	33.7 (1.1)	50.9 (0.5)	18.6 (1.1)	95.0	95.0	25.0
NOR	37	34.5 (1.1)	52.0 (0.5)	18.7 (1.1)	85.7	100.0	33.3
NAR	38	27.7 (1.2)	45.7 (0.6)	18.9 (1.2)	95.0	75.0	0.0
NAR	39	27.8 (1.1)	44.6 (0.5)	17.8 (1.1)	95.2	100.0	4.8
NAR	40	29.7 (1.2)	46.4 (0.6)	17.8 (1.2)	94.4	77.8	0.0
NAR	41	30.9 (1.2)	46.4 (0.6)	16.4 (1.2)	94.7	84.2	0.0
SNC	42	21.5 (1.2)	44.7 (0.6)	24.1 (1.2)	77.8	100.0	5.6
SNC	43	24.1 (1.1)	45.6 (0.5)	22.7 (1.2)	85.0	95.0	0.0
SNC	44	27.2 (1.3)	44.2 (0.6)	17.9 (1.3)	68.4	73.7	5.3
SNC	45	29.7 (1.2)	45.8 (0.6)	17.0 (1.2)	88.9	88.9	0.0
SNC	46	33.7 (1.3)	51.3 (0.6)	18.9 (1.3)	100.0	100.0	13.3
TYG	47	21.9 (1.2)	42.3 (0.5)	21.6 (1.2)	89.5	94.7	0.0
TYG	48	24.9 (1.1)	45.5 (0.5)	21.6 (1.1)	100.0	100.0	5.0
TYG	49	26.8 (1.2)	46.1 (0.5)	20.3 (1.2)	89.5	94.7	0.0
TYG	50	28.3 (1.2)	45.4 (0.6)	18.1 (1.2)	100.0	100.0	0.0
TYG	51	28.7 (1.3)	46.7 (0.6)	19.0 (1.3)	100.0	92.9	0.0
TYG	52	28.8 (1.1)	47.3 (0.5)	19.5 (1.1)	81.0	100.0	4.8
TYG	53	29.4 (1.1)	44.0 (0.5)	15.6 (1.2)	85.0	95.0	0.0
TYG	54	29.5 (1.2)	48.3 (0.5)	19.8 (1.2)	76.2	95.2	4.8
TYG	55	29.6 (1.2)	46.8 (0.6)	18.2 (1.2)	88.9	88.9	0.0
TYG	56	29.7 (1.2)	48.1 (0.6)	19.3 (1.3)	68.4	94.7	0.0
TYG	57	29.7 (1.1)	45.9 (0.5)	17.2 (1.1)	95.0	95.0	0.0
WES	58	24.1 (1.4)	44.7 (0.7)	21.8 (1.4)	85.7	85.7	14.3
WES	59	30.6 (1.2)	48.3 (0.6)	18.6 (1.2)	94.4	88.9	11.1
WES	60	32.7 (1.2)	47.1 (0.5)	15.5 (1.2)	94.4	100.0	0.0
